# Assessment of positive psychological functioning among Chinese university students: Revision and psychometric properties of a psychological capital scale

**DOI:** 10.1371/journal.pone.0323731

**Published:** 2025-06-13

**Authors:** Yanlan Chen, Xiaowei Feng, Syed Kamaruzaman Syed Ali, Hutkemri Zulnaidi

**Affiliations:** 1 School of Physical Education, Guangdong University of Education, Guangdong, China; 2 Faculty of Physical Education, Hainan Normal University, Haikou, Hainan, China; 3 Department of Educational Foundation and Humanities, Faculty of Education, University of Malaya, Kuala Lumpur, Malaysia,; 4 Department of Mathematics and Science Education, Faculty of Education, University of Malaya, Kuala Lumpur, Malaysia; Northwestern University Feinberg School of Medicine, UNITED STATES OF AMERICA

## Abstract

Psychological capital serves as a psychological asset facilitating personal growth and enhanced performance. It aids individuals in navigating life’s myriad challenges and adversities, promoting psychological well-being and adaptability. Despite the widely recognized importance of psychological capital in enhancing competitiveness and work performance, existing assessment tools, such as the PCQ-24 developed by Luthans et al. (2007), have not been adequately validated in the Chinese university student population. To explore the factor structure of the Psychological Capital Questionnaire (PCQ-24) developed by Luthans et al., the researchers employed Principal Component Analysis (PCA) and Exploratory Factor Analysis (EFA), extracting factors with eigenvalues greater than 1. The results revealed a six-factor structure, which is inconsistent with the original four-factor structure of the PCQ-24 measurement tool. Therefore, Luthans’ psychological capital scale is not suitable for university students. Additionally, the indigenous scale developed by Chinese researchers Ke Jianglin et al (2009) is more suitable for organizational employees rather than university students. Hence, it is essential to revise and validate a psychological capital measurement tool appropriate for Chinese university students. To fill this gap, the researchers aimed to revise and validate a psychological capital scale suitable for Chinese university students based on the four structural dimensions proposed by Luthans et al. In this study, a large-scale survey of Chinese university students (N = 2780) was conducted, and SPSS 26.0 and AMOS 24.0 were used to statistically analyze the data and assess the psychometric properties of the Revised Mental Capital Scale. The results indicate that the revised psychological capital scale meets the psychometric requirements. The study concluded that the psychological capital scale revised and validated by the researcher can be used as an instrument to measure and assess the psychological capital of university students.

## Introduction

The concept of psychological capital was first introduced in industrial organizational settings and is considered a critical success factor for competitiveness and productivity [1]. With accelerated globalization, rapid economic growth, and rising education levels, the study of the mental health and positive psychological functioning of university students, as the backbone of future society, has become particularly important. In the university student population, mental health not only affects their academic performance and quality of life, but also relates to their future social adaptability and career development [2]. However, university students as a group are facing multiple challenges such as academic pressure and competition for employment, so it is of great practical significance to explore factors that can help improve their mental health.

Positive psychological functioning, as an important factor in an individual’s ability to cope with challenges and stress and maintain mental health, has received much attention in recent years [3]. Research has shown that positive psychological functioning can help university students manage stress more effectively, enhance academic achievement, and increase life satisfaction [2]. Based on this background, as a combination of positive psychological resources and psychological abilities, including optimism, self-efficacy, hope and resilience, psychological capital is acknowledged as a fundamental factor for individuals to navigate hardships and challenges [1]. Not only does psychological capital play an important role in the workplace environment, but it is also becoming an important theoretical framework for the study of university students’ mental health and development in the field of education.

In recent years, growing attention has been directed towards the positive effects of psychological capital on individuals’ academic outcomes within educational settings, in recent years, the beneficial influence of psychological capital on individuals’ academic achievements in educational settings has also received much attention [4–8]. A substantial body of research indicates a close relationship between levels of psychological capital (PsyCap) and the psychological well-being of university students [9–12]. University students possessing higher levels of psychological capital are more likely to exhibit increased subjective well-being and psychological happiness [13–15], along with lower levels of anxiety and depression [16–22]. Consequently, the level of psychological capital in university students not only significantly influences individual psychological well-being but also exerts positive effects on their academic and career development.

Despite the fact that psychological capital is crucial to the mental health and development of university students, there are still very few reliable and valid instruments to accurately measure this characteristic. Current psychological capital assessment tools for the Chinese university student population still need to be improved and developed. The applicability and reliability of widely used psychological capital scales abroad, such as the PCQ-24 created by Luthans et al. (2007) [10], have not yet been fully validated in the Chinese university student population. Moreover, the scale has been studied mainly with staff and managers [23–26] and has limited applicability in the university student population. Although Ke Jianglin et al. (2009) [27] developed a local psychological capital scale that fits the Chinese context, it is still mainly applicable to organizational employees and not fully applicable to the university student population.

Therefore, based on the characteristics of Chinese university students, this study aims to revise and validate the psychological capital scale applicable to this group of students. We adopted the four structural dimensions of psychological capital proposed by Luthans et al. With the aim of comprehensively assessing the level of positive psychological functioning of university students, and providing a scientific basis and an effective tool for the promotion of university students’ psychological health and overall development. In this study, university students in mainland China were used as subjects. The researchers used internal consistency analysis to evaluate the reliability of Luthans’ PCQ-24, the findings indicated that the reliability of the PCQ-24 was unsatisfactory for measuring the psychological capital of university students. Therefore, it is necessary to revise the PCQ-24 into a suitable psychological capital scale for university students and test its psychometric properties. Therefore, we further revised the PCQ-24 scale and used exploratory factor analysis to assess its psychometric properties in the Chinese cultural context in order to construct a psychological capital scale more suitable for Chinese university students.

## Materials and methods

This study is a cross-sectional study, and all participants have signed written informed consent forms. Additionally, this study has been approved by the Ethics Committee of the University of Malaya (No: UM.TNC2/UMREC_2731).

### Participants

In this study, students from public universities in Jiangxi Province, China, were selected as the survey participants. Considering that there are a total of 11 districts in Jiangxi Province, 11 public universities were randomly selected as the source of participants. Human participants were recruited for this study to distribute questionnaires to obtain research data. The start date of the recruitment period was from October 2023 to December 2023. No minors will be involved in this study, all participants will be adults over the age of 18 and all participants will have signed an informed consent form. This research involved the participation of 3,270 university students. Participants from Nanchang completed surveys on-site, while participants from other regions completed surveys online. A total of 3,270 questionnaires were distributed on-site and online. After screening, 2,780 valid surveys were obtained, resulting in an effective response rate of 85%. These data provide a substantial sample basis for the study and offer critical support for the scientific rigor and reliability of the research findings.

To ensure the representativeness and scientific validity of the sample for this study, the researchers conducted a detailed demographic survey covering important information such as gender, grade level and place of birth of the participants. The sample consisted of 1,145 male students (41.2% of the total) and 1,635 female students (58.8% of the total). In addition, 1,212 students, or 43.6% of the total, were from urban areas and 1,568 students, or 56.4% of the total, were from rural areas. In the survey, researchers balanced the gender ratio of participants to ensure a relatively even distribution of males and females in the sample, reflecting the actual demographic composition of university students. Additionally, researchers paid attention to the distribution of participants across different grade levels, ensuring inclusion of students from various stages of university education, ranging from freshmen to seniors, to comprehensively capture the state of psychological capital across different stages. Furthermore, researchers specifically focused on participants’ place of birth information, including the distribution between urban and rural areas. Through these detailed surveys and analyses, researchers ensured the diversity and representativeness of the sample, enhancing the generalizability and applicability of the research findings. This meticulous approach in demographic investigation and sampling strategy strengthens the validity and reliability of the study’s conclusions regarding psychological capital among university students.

### Instruments and measures

This study used the revised Subjective Well-Being Scale by Pontin et al. which focuses on assessing an individual’s subjective experience of their physical health, psychological health, independence, social relationships, environments, and spiritual quality of life [28].

This study utilized the Subjective Well-Being Scale as a criterion measure to assess the validity of the Psychological Capital Scale for university students. The scale consists of a total of 24 self-report items. A Likert 5-point scale (1 = not at all-5 = extremely) was used. The Subjective Well-Being Scale demonstrated a high level of internal consistency, with a Cronbach’s alpha coefficient of 0.768, indicating good measurement reliability within the sample. Therefore, this scale was utilized as a criterion measure in this study. Moreover, the multidimensional structure of the scale was confirmed by a validated factor analysis (CFA), indicating that it can effectively differentiate different dimensions of subjective well-being. In addition, the validity and reliability of the subjective well-being scale have been widely validated in the Chinese context.

#### Back-Translation and language refinement.

First, the forward translation was conducted by translating the revised original Chinese questionnaire into English. To ensure the accuracy and cultural appropriateness of the translation, two professional translators with extensive experience and expertise in the relevant field were invited. This was done to ensure that the English version of the questionnaire is comprehensible and effective in an English-speaking cultural context. After the translation was completed, the English version of the questionnaire underwent grammatical and lexical refinement. This process involved correcting any potential grammatical errors, improper word choices, or unclear expressions to enhance the language quality and fluency of the questionnaire.

Next, a back-translation was performed to verify the accuracy and consistency of the translation. Two additional senior English translation experts, who were not involved in the forward translation, were invited to translate the refined English questionnaire back into Chinese. The purpose of this step was to evaluate whether any loss of original meaning or distortion occurred during the translation process.

Then, a comparison and revision were carried out. The researchers meticulously compared the back-translated Chinese questionnaire with the original Chinese version. By comparing the content of each item, any potential discrepancies or misunderstandings were identified. Based on the comparison results, the English questionnaire was revised as necessary to ensure consistency and equivalence across different languages and cultural contexts. The final version of the questionnaire, obtained after the revisions, will serve as the formal psychological capital measurement tool for this study.

### Statistical analysis

Statistical analysis of the study was conducted using SPSS version 26.0 and AMOS version 24.0. This study employed two independent subsamples for data analysis. Subsample 1 (N = 1520) was used for Exploratory Factor Analysis (EFA), while Subsample 2 (N = 1260) was used for Confirmatory Factor Analysis (CFA). Descriptive statistical analysis was initially conducted on the data, followed by an examination of item characteristics to assess their suitability. Additionally, we analyzed the factor structure of the Psychological Capital Questionnaire (PCQ-24) developed by Luthans et al [10]. A pilot study was conducted with N = 420 participants employing exploratory factor analysis with varimax rotation using principal component analysis. By extracting psychological measurement criteria with eigenvalues greater than 1, a factor structure comprising 6 factors was identified, which diverged from the original 4-dimensional structure of the PCQ-24 measurement tool, as depicted in Table 1. Consequently, this extensive analysis indicates that the PCQ-24 version of the Psychological Capital Questionnaire is not applicable to the university student population.

**Table 1:** Results of factor analysis for explained total variance in the pilot study.

	Initial Eigenvalues			Extraction Sums of Squared Loadings			Rotation Sums Squared Loadings		
component	Total	% of Variance	Cumulative%	Total	% of Variance	Cumulative%	Total	% of Variance	Cumulative%
1	14.271	40.775	40.775	14.271	40.775	40.775	5.043	14.409	14.409
2	2.451	7.003	47.778	2.451	7.003	47.778	4.154	11.870	26.278
3	2.069	5.910	53.689	2.069	5.910	53.689	3.967	11.333	37.612
4	1.831	5.230	58.919	1.831	5.230	58.919	3.533	10.093	47.705
5	1.530	4.372	63.291	1.530	4.372	63.291	2.842	8.121	55.826
6	1.208	3.453	66.743	1.208	3.453	66.743	2.562	7.320	63.146

Furthermore, it is important to note that the PCQ-24 questionnaire consists of 24 items, among which 19 items contain keywords related to “at work” or “on the job.” This design implies that the scale is more suitable for application with groups who have actual work experience, such as employees in organizations. Considering that the participants in this study are university students who mostly lack formal work experience, using such a scale may limit the accurate assessment of their work attitudes and behaviors. This suggests the need for a scale more tailored to the actual circumstances and experiences of university students to better understand and evaluate their levels of psychological capital. Based on this consideration, researchers revised a psychological capital scale suitable for Chinese university students to ensure the effectiveness and accuracy of the assessment.This adaptation aimed to address the unique context of university students and provide a more appropriate instrument for assessing their psychological capital levels.

Descriptive statistics were initially conducted on in this study for two sub-samples. The data from subsample 1 was subjected to the KMO measure and Bartlett’s sphericity test to examine the validity of the scale items. Principal component analysis was then performed, using an eigenvalue greater than 1 as the criterion for factor extraction. Furthermore, the researchers conducted an exploratory factor analysis on subsample 2 to validate the factor structure of the revised Psychological Capital scale. This comprehensive approach aimed to rigorously assess the effectiveness and validity of the revised scale through statistical analyses across both subsamples.

The researchers employed seven indicators to evaluate the model’s adaptability, including Root Mean Square Error of Approximation (RMSEA), the ratio of chi-square value to degrees of freedom (χ2/df), Goodness-of-Fit Index (GFI), Comparative Fit Index (CFI), Tucker-Lewis Index (TLI), Incremental Fit Index (IFI), and Normed Fit Index (NFI). The specific evaluation criteria were as follows: if RMSEA ≤ 0.08 and χ2/df ≤ 5.00, and if GFI, CFI, NFI, IFI, and TLI ≥ 0.90, then it indicates a good model fit [27].

In this study, the validity of the university students’ psychological capital questionnaire was assessed using Pearson’s correlation analysis, and the university students’ subjective well-being scale was chosen as the validity scale. Additionally, researchers used Cronbach’s alpha coefficient to measure the internal consistency reliability of the revised version of the Psychological Capital scale, which demonstrated high reliability in psychological measurement testing [29]. During the evaluation of the revised Psychological Capital scale, researchers calculated factor loadings, composite reliability (CR), and average variance extracted (AVE). The results indicated that the factor loadings of the revised scale were >0.6, CR values were >0.5, and AVE was > 0.8. These indicators collectively demonstrate strong reliability and validity of the revised Psychological Capital scale. Through Pearson correlation analysis, researchers assessed the correlation between the Psychological Capital Questionnaire and the subjective well-being scale to validate the effectiveness of the Psychological Capital Questionnaire as a criterion measure. Furthermore, Cronbach’s alpha coefficient was utilized to evaluate the internal consistency reliability of the revised Psychological Capital scale, revealing high consistency and reliability in measuring psychological capital among university students.

## Results

### Sample description

This study recruited a total of 2,780 Chinese university students, consisting of 1,145 males (41.2%) and 1,635 females (58.8%). Participants were undergraduate students from 11 regions in Jiangxi Province, China. Among them, the exploratory factor analysis subsample consisted of N = 1,520 participants, and the confirmatory factor analysis subsample consisted of N = 1,260 participants.

### Descriptive statistics

The Psychological Capital scale comprises four dimensions: self-efficacy, hope, resilience, and optimism. In this investigation, the items of the four dimensions of the Psychological Capital Scale were statistically analyzed, including calculation of mean, standard deviation, and tests for normality (Table 2). To evaluate the data’s normality, researchers utilized skewness and kurtosis measurements as proposed by George and Mallery (2016) [30]. For data to be considered normally distributed, the absolute values of skewness and kurtosis are expected to be within the range of -1.96 and +1.96. During the normality assessment, skewness and kurtosis were converted into Z-scores using the formula: Skewness Z-score is calculated by dividing the skewness value by the standard error, while kurtosis Z-score is obtained by dividing the kurtosis value by the standard error. If these Z-scores lie between ±1.96, it indicates that the research data follow a normal distribution.

**Table 2:** Normality test.

Item	Item Score		Skewness and Kurtosis	
	M	SD	S	K
Sell-efficacy	19.36	3.114	-0.807	-1.315
Hope	18.71	3.542	-0.731	-1.235
Resilience	19.53	3.519	-0.387	-0.803
Optimistic	19.71	3.087	0.915	-1.412

The study conducted skewness and kurtosis tests on the four variables, and the resulting Z-scores fell within the range of -1.96 and +1.96, confirming the assumption of normal distribution for the data. The participants’ average scores were as follows: self-efficacy score of 19.36 (standard deviation: 3.114), hope score of 18.71 (standard deviation: 3.542), resilience score of 19.53 (standard deviation: 3.519), and optimism score of 19.71 (standard deviation: 3.087).

### Item analysis

#### Exploratory factor analysis.

To evaluate the validity of the scale items, an exploratory factor analysis (EFA) was conducted on subsample 1 data for the items of the Psychological Capital questionnaire. The findings revealed that the Kaiser-Meyer-Olkin (KMO) measure yielded a value of 0.834, suggesting that the data has good sampling adequacy, surpassing the threshold of 0.7, which is suitable for factor analysis. Additionally, Bartlett’s test of sphericity was performed, indicating a chi-square value of approximately 2252.732, with a corresponding significance level (Sig) of 0.000 (Item < 0.001), indicating significant correlations among the variables in the dataset. These findings collectively indicate that the employed Psychological Capital questionnaire possesses good structural validity and meets the basic requirements for conducting factor analysis. The results of the factor analysis are depicted in Table 3.

**Table 3:** Results of factor analysis for explained total variance of the formal questionnaire.

	Initial Eigenvalues			Extraction Sums of Squared Loadings			Rotation Sums Squared Loadings		
component	Total	% of Variance	Cumulative%	Total	% of Variance	Cumulative%	Total	% of Variance	Cumulative%
1	9.120	45.598	45.598	9.120	45.598	45.598	3.947	19.735	19.735
2	1.948	9.738	55.336	1.948	9.738	55.336	3.608	18.042	37.776
3	1.721	8.603	63.938	1.721	8.603	63.938	3.461	17.307	55.083
4	1.313	6.566	70.505	1.313	6.566	70.505	3.084	15.421	70.505

Table 3 outlines the factor extraction process for the formal Psychological Capital questionnaire. Principal component analysis (PCA) was utilized, with the criterion of eigenvalues exceeding 1 for factor extraction, resulting in the successful extraction of four factors. The cumulative contribution rate reached 70.505%, exceeding the 70% threshold, indicating that the factor analysis yielded highly satisfactory results. This alignment demonstrates that the revised Psychological Capital questionnaire used in the study fully conforms to the conceptual framework of Psychological Capital’s four-dimensional structure as proposed by Luthans. The results show that: Component 1 comprises Hope 2, Hope 3, Hope 4, Hope 1, and Hope 5, which aligns with the predetermined hope sub-dimension. Component 2 consists of Self-efficacy 5, Self-efficacy 1, Self-efficacy 2, Self-efficacy 3, and Self-efficacy 4, corresponding exactly to the predetermined self-efficacy sub-dimension. Component 3 includes Optimism 1, Optimism 2, Optimism 3, Optimism 5, and Optimism 4, matching the predetermined optimism sub-dimension. Component 4 is composed of Resilience 4, Resilience 2, Resilience 5, Resilience 3, and Resilience 1, in line with the predetermined resilience sub-dimension. These findings from the component matrix further validate the strong structural validity of the revised Psychological Capital questionnaire.

#### Validation factor analysis.

**Fig 1:**
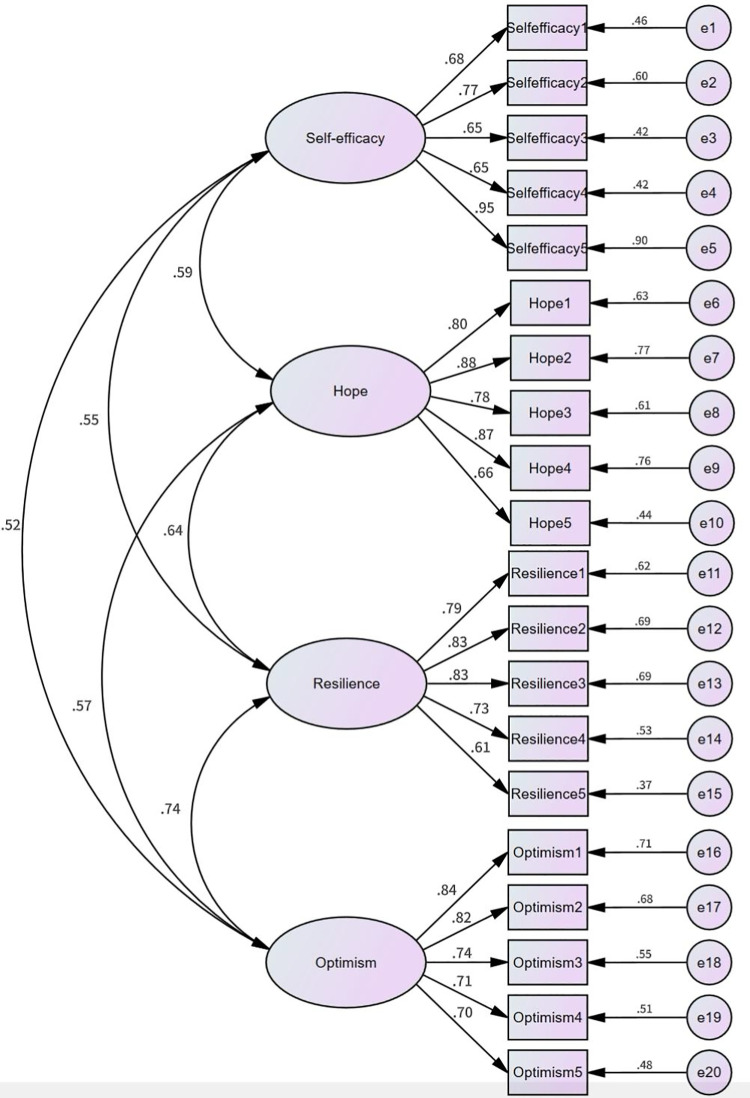
Confirmatory analysis path diagram of the psychological capital structure in university students.

AMOS 24.0 was employed to perform a validation factor analysis of subsample 2 to verify the reasonableness of the psychological capital structure. According to the outcomes of the factor validation analysis, seven validation model fit indices for the measurement models of university students’ psychological capital were obtained. Considering the aforementioned results, the metrics of the validated factor analysis (CFA) are shown to be: χ2/df = 2.880, SRMR = 0.067, GFI = 0.901, CFI = 0.909, and IFI = 0.910.According to Hair et al (2021) [31], the fit metrics in the Structural Equation Modeling (SEM) should meet the following criteria: χ2/df ≤ 5.00, RMSEA ≤ 0.08, and CFI, GFI, and IFI ≥ 0.90, suggesting that the model adequately fits the data. In this study, the various validation fit indices basically met the requirements for a well-fitting model. Although the NFI and TFI values in the model were slightly lower than 0.90, they still exceeded 0.80 and were close to the edge of 0.90. This indicates that the model demonstrated a satisfactory overall fit. Therefore, the above indicators show that the model has achieved more satisfactory results in the validation of the psychological capital structure of university students. The standardized path coefficient diagram of the model in question is detailed in Fig 1.

The findings in Table 4 illustrate the reliability and validity assessment of the revised psychological capital scale. In terms of reliability, the Composite Reliability (CR) value exceeding 0.5 indicates that the scale factors exhibit strong internal consistency and stability and can effectively reflect the concepts measured. In addition, in terms of validity, the Average Variance Extracted (AVE) is greater than 0.8, which indicates that the factors in the scale are able to explain the variance of the variables they measure well and have good convergent validity. Taken together, these results indicate that the revised psychological capital scale exhibits good properties in terms of reliability and validity and can be reliably used to measure concepts and variables related to psychological capital.

**Table 4:** Variable measurements and index results.

Variables	Item	Factor loading	Cronbach’Alpha	AVE	CR
Self-efficacy	I am confident that I can pass all the exams during my university year	0.676	0.845	0.560	0.861
	I am confident that I can handle the relationship with teachers and classmates well	0.775			
	I have the confidence to complete the tasks assigned by the teacher	0.651			
	I have the confidence to accomplish the learning goals I set for myself	0.651			
	I have the confidence to accept new things more easily than others	0.946			
Hope	I am full of hope for the future work	0.796	0.896	0.643	0.899
	I am hopeful of completing my studies successfully	0.880			
	I am hopeful about my life goals	0.780			
	I am very hopeful about my personal career development	0.874			
	I am very hopeful about my professional future	0.660			
Resilience	I can persist in seeking solutions to difficulties	0.790	0.870	0.580	0.872
	In difficult situations, I can insist on proactively trying different strategies	0.831			
	When I encounter setbacks, I can learn from my experience and keep going	0.829			
	When I encounter a difficult problem, I can insist on using my initiative until I learn it	0.728			
	To accomplish the goal, I can insist on working hard for it.	0.605			
Optimism	I can face setbacks optimistically and always keep a positive attitude	0.840	0.872	0.584	0.875
	I can face failure with optimism and tell myself not to give up lightly	0.823			
	I can face life with optimism and be happy every day	0.739			
	I can be optimistic about uncertain outcomes and always look on the bright side	0.712			
	I can be optimistic about my teachers’ guidance and keep improving myself	0.695			

#### Criterion validity.

This study assessed the validity of the validity scale between the Psychological Capital Questionnaire and the Subjective Well-Being Scale for university students using Pearson’s correlation analysis. Among the many factors affecting university students’ subjective, well-being, self-efficacy [32–35], optimism [2,36–38], and other factors have been found to have a positive impact on subjective well-being. Therefore, this study aimed to validate the psychological capital scale for university students, which is significant in the university student population. Based on the actual characteristics of university students, we analyzed the correlation between the scores of the psychological capital questionnaire and the scores of subjective well-being of university students. The study findings are presented in Table 5 below. This analysis provides deeper insights into the correlation between psychological capital and subjective well-being, and thus assess the validity and usefulness of the psychological capital questionnaire in the university student population.

**Table 5:** Correlation between psychological capital and subjective well-being.

Scale	Goal Pursuit	Life Satisfaction	Positive Emotion	Quality of Life	Sense of Meaning	Total Subjective Well-Being Score
Self- efficacy	.399**	.297**	.314**	.353**	.337**	.481**
Hope	.401**	.287**	.311**	.359**	.411**	.500**
Resilience	.403**	.294**	.326**	.398**	.759**	.610**
Optimism	.483**	.423**	.446**	.378**	.445**	.617**
Total Psycap Score	.523**	.402**	.432**	.464**	.615**	.688**

Table 5 explores the correlation between psychological variables measured by the psychological capital questionnaire for university students and individual subjective well-being, with subjective well-being as the criterion. The findings suggest that all four dimensions of psychological capital are positively correlated with the five dimensions of subjective well-being. The Pearson correlation coefficient between the total scores of psychological capital and subjective well-being is 0.688 (P < 0.01), suggesting a significantly positive correlation between psychological capital and subjective well-being.

### Reliability test

This questionnaire consists of four dimensions of psychological capital, namely: self-efficacy, hope, resilience, and optimism. The Cronbach’s Alpha coefficients for these four dimensions and the overall scale all exceed 0.8, indicating high reliability and meeting the requirements for the reliability of psychological scales. Therefore, the revised Psychological Capital Scale for university students demonstrates high reliability. The results of the reliability analysis are as follows:

The results presented in Table 6 demonstrate that the Cronbach’s Alpha coefficient for the revised Psychological Capital Questionnaire is 0.935. The Cronbach’s Alpha coefficients for the sub-dimensions, specifically self-efficacy, hope, resilience, and optimism, are 0.845, 0.896, 0.870, and 0.872, correspondingly. Therefore, the questionnaire exhibits desirable reliability.

**Table 6:** Results of Cronbach’s Alpha test for each sub-dimension of the scale.

	Cronbach’s Alpha	N of Item
Self-efficacy	0.845	5
Hope	0.896	5
Resilience	0.870	5
Optimism	0.872	5
Psychological Capital	0.935	20

## Discussion

This study revised and validated a psychological capital scale suitable for Chinese university students, aiming to gain a deeper understanding of their positive psychological functioning. Previous research has shown that psychological capital is a positive psychological strength demonstrated by individuals throughout their growth [39–42], which helps enhance subjective well-being [14,43,44]. It possesses a certain level of stability, while also being subject to change and development. Among Chinese university students, psychological capital plays a significant role in coping with academic pressure [13,19], interpersonal relationships [45–47], and career planning [48–50]. Therefore, revising and validating a psychological capital scale for Chinese university students is crucial for gaining insights into and evaluating their positive psychological functioning.

Through a literature review and Luthans’ proposed four-dimensional structure, we revised the psychological capital scale and validated its structural validity through item analysis, exploratory factor analysis, and confirmatory factor analysis. The results indicate that university students’ psychological capital consists of four factors: self-efficacy, hope, resilience, and optimism. The revised scale demonstrates good structural validity, criterion validity, and reliability. It effectively measures the psychological capital state of university students and has good applicability within the Chinese university student population. Additionally, it contributes to better promoting the psychological health of Chinese university students and provides important references for future educational practices.

## Conclusions

This study aims to revise and validate a psychological capital scale suitable for Chinese university students and explore its application and significance in assessing positive psychological functioning among university students. By strictly adhering to the fundamental standards of scale revision, we successfully revised the psychological capital scale and validated its structure and effectiveness through item analysis, exploratory factor analysis, and confirmatory factor analysis. The results indicate that university students’ psychological capital primarily consists of four core factors: self-efficacy, hope, resilience, and optimism, which align with Luthans’ proposed four-dimensional structure of psychological capital. The findings from exploratory and confirmatory factor analyses demonstrate that the revised psychological capital scale exhibits good structural validity and criterion validity, effectively measuring university students’ psychological capital status with high reliability and stability.

Further exploring the applicability and utility of the scale within the Chinese university student population, researchers found that the psychological capital scale can reveal the positive psychological functioning status of Chinese university students, providing important scientific foundations and effective strategies for psychological health education and intervention. The scale serves as an effective tool for assessing university students’ psychological health, aiding schools and social institutions in designing targeted psychological health education and support programs to promote comprehensive development and growth.

The revision and validation of a psychological capital scale suitable for Chinese university students are crucial tools for gaining deep insights into and assessing university students’ positive psychological functioning. The use of this tool will provide researchers with more information and insights to conduct deeper studies on university students’ psychological capital and its impact on their lives and career development. Through this study, we not only deepen our understanding of university students’ psychological capital but also provide more evidence for education policy-making and psychological health promotion, offering theoretical support and practical guidance for enhancing university students’ psychological health. Further exploration of the relationship between psychological capital and academic achievement., career adaptability, and life satisfaction to provide deeper insights and recommendations for university student mental health and educational practice.

### Limitations of the study

There are several methodological limitations in the results of this study. Firstly, similar to most survey research, this study has its own constraints. The revised psychological capital scale was designed based on the psychological characteristics of Chinese university students. raising doubts about its applicability in other countries and cultural contexts. Future research should focus on validating the questionnaire’s effectiveness in different cultural and national groups to further confirm its universality and applicability.

Secondly, the sample used in this study consisted of Chinese university students, but this cannot represent all university student populations. Students from different disciplines, grades, and geographical locations may possess different psychological characteristics. For a thorough grasp of the psychological capital among university students, future research can expand the sample coverage to better represent global university student groups, thereby improving the universality and generalizability of the research results.

Additionally, the revised questionnaire primarily relied on self-reporting by participants, which may introduce subjectivity and recall bias. To enhance the credibility and objectivity of the findings, objective measures can be used in future studies. For instance, observation and physiological indicators to supplement and verify self-reported data, thus providing a more comprehensive assessment of university students’ psychological capital status.

Therefore, despite achieving certain successes in revising and validating the psychological capital scale, this study still has methodological limitations. Future research should focus on improving and refining these limitations to enhance the scientific rigor, universality, and practicality of the psychological capital scale, thereby providing a more effective scientific basis and strategies for assessing and intervening in university students’ psychological health.

### Research implications and future directions

The revised psychological capital questionnaire in this study emphasizes the importance of group specificity, providing strong support for the application of psychological research across different cultural and temporal contexts. The revised questionnaire not only more accurately reflects and measures the psychological capital characteristics of Chinese university students but also better adapts to the unique challenges faced by contemporary students. Theoretically, the revised questionnaire promotes the localization of psychological capital theory by capturing the psychological capital features of Chinese university students, thus providing a foundation for further exploration of the applicability of psychological capital in different cultural contexts. Practically, the revised questionnaire offers an effective tool for developing personalized psychological capital intervention strategies. Additionally, this questionnaire can serve as a unified measurement tool for global psychological capital research, fostering cross-cultural comparative studies and expanding the international perspective on psychological capital theory and practice.

Future research should further conduct cross-national and cross-cultural validation to confirm the universality and consistency of the questionnaire, thereby providing crucial support for international comparative studies. Additionally, it can delve deeper into key factors influencing students’ psychological capital, such as educational environment, social support, and individual experiences, to comprehensively understand the formation mechanisms of psychological capital in university students. Future studies could also develop a multidimensional psychological capital scale to improve measurement precision and explore effective strategies for cultivating and intervening in psychological capital, enhancing students’ self-efficacy, hope, resilience, and optimism, and helping them better cope with stress and challenges, thereby promoting psychological well-being and overall development. Furthermore, the questionnaire can be used for individual psychological assessments, providing a basis for personalized psychological counseling, and through regular evaluations of the impact of psychological capital on students’ mental health and academic performance, it will further advance the development of psychological capital theory and practice.

## Supporting information

S1 Appendices Revised psychological capital measurement instrument.
(DOCX)

S1 File Inclusivity in global research.
(DOCX)
