# Supplementary material for: Assessment of positive psychological functioning among Chinese university students: Revision and psychometric properties of a psychological capital scale
Source: PLoS One. 2025 Jun 13;20(6):e0323731. doi: 10.1371/journal.pone.0323731 (PMC12165361; doi:10.1371/journal.pone.0323731)
Supplement: S1 Appendices — Revised psychological capital measurement instrument. (DOCX) [file pone.0323731.s001.docx]

**Appendices：****Revised Psychological Capital Measurement Instrument**

|  | ITEMS | Perfectly in line with | Basically in line with | Uncertain | Basic non-compliance | Not at all consistent with |
| --- | --- | --- | --- | --- | --- | --- |
| 1 | I am confident to pass all the exams during my university years |  |  |  |  |  |
| 2 | I have the confidence to handle the relationship with teachers and classmates well |  |  |  |  |  |
| 3 | I have the confidence to complete the tasks assigned by the teacher |  |  |  |  |  |
| 4 | I have the confidence to accomplish the learning goals I set for myself |  |  |  |  |  |
| 5 | I have the confidence to accept new things more easily than others |  |  |  |  |  |
| 6 | I am full of hope for my future work |  |  |  |  |  |
| 7 | I am hopeful of successfully completing my studies |  |  |  |  |  |
| 8 | I’m hopeful about my life goals |  |  |  |  |  |
| 9 | I am very hopeful about my personal career development |  |  |  |  |  |
| 10 | I’m hopeful about my professional future |  |  |  |  |  |
| 11 | In the face of difficulties, I can persist in seeking solutions |  |  |  |  |  |
| 12 | In difficult situations, I can insist on proactively trying different strategies |  |  |  |  |  |
| 13 | When I encounter setbacks, I can learn from my experiences and keep going |  |  |  |  |  |
| 14 | When I encounter a difficult problem, I can insist on using my initiative and motivation until I learn it |  |  |  |  |  |
| 15 | In order to accomplish the goal, I can keep working hard for it. |  |  |  |  |  |
| 16 | I can face setbacks with optimism and always keep an upward mindset. |  |  |  |  |  |
| 17 | I can face failure with optimism and tell myself not to give up lightly. |  |  |  |  |  |
| 18 | I can face life with optimism and be happy every day |  |  |  |  |  |
| 19 | I can be optimistic about uncertain outcomes and always look on the bright side |  |  |  |  |  |
| 20 | I can be optimistic about my teachers’ guidance and keep improving myself |  |  |  |  |  |
